# ﻿Exploring diversity within the genus *Tulostoma* (Basidiomycota, Agaricales) in the Pannonian sandy steppe: four fascinating novel species from Hungary

**DOI:** 10.3897/mycokeys.100.112458

**Published:** 2023-11-29

**Authors:** Péter Finy, Mikael Jeppson, Dániel G. Knapp, Viktor Papp, László Albert, István Ölvedi, Károly Bóka, Dóra Varga, Gábor M. Kovács, Bálint Dima

**Affiliations:** 1 Department of Plant Anatomy, Institute of Biology, Eötvös Loránd University, Pázmány Péter sétány 1/C, Budapest 1117, Hungary Eötvös Loránd University Budapest Hungary; 2 Hungarian Mycological Society, Könyves Kálmán krt. 40. Budapest 1087, Hungary Hungarian Mycological Society Budapest Hungary; 3 University of Gothenburg, Biological and Environmental Sciences, P.O. Box 461, SE-40530 Göteborg, Sweden University of Gothenburg Göteborg Sweden; 4 Department of Forestry and Wood Technology, Linnaeus University, Växjö, Sweden Linnaeus University Växjö Sweden; 5 Department of Botany, Hungarian University of Agriculture and Life Sciences, Villányi út 29–43, H-1118 Budapest, Hungary Hungarian University of Agriculture and Life Sciences Budapest Hungary

**Keywords:** Gasteroid, hot spot, molecular systematics, Pannonian inland sand dune thicket, phylogeny, taxonomy, Tulostomataceae

## Abstract

Steppe vegetation on sandy soil in Hungary has recently been revealed as one of the hot spots in Europe for the stalked puffballs (genus *Tulostoma*). In the framework of the taxonomic revision of gasteroid fungi in Hungary, four *Tulostoma* species are described here as new to science: *T.dunense*, *T.hungaricum*, *T.sacchariolens* and *T.shaihuludii*. The study is based on detailed macro- and micromorphological investigations (including light and scanning electron microscopy), as well as a three-locus phylogeny of nrDNA ITS, nrDNA LSU and *tef1-α* sequences. The ITS and LSU sequences generated from the type specimen of *T.cretaceum* are provided and this resolved partly the taxonomy of the difficult species complex of T.aff.cretaceum.

## ﻿Introduction

The genus *Tulostoma* was erected by Persoon (1794, 1801) encompassing two species, *T.brumale* and *T.squamosum*. Several new species have since been added from all continents, except Antarctica. In a monograph of the genus based on type studies, Wright (1987) accepted 139 species worldwide. Later studies generally confirmed those species concepts, nevertheless reduced some of the species to synonymy (e.g. Moreno et al. (1992, 1997); Altés et al. (1999); Jeppson et al. (2017)). With the introduction of molecular methods in taxonomy, unexpected species diversity has been detected and new, formerly unknown species have been described. In Europe, Jeppson et al. (2017) suggested Mediterranean grassland regions of the Iberian Peninsula, as well as steppe habitats in East Central Europe, as hot-spot areas for species diversity in *Tulostoma*. Jeppson et al. (2017) described two novel species with type localities in Central Hungary (*T.grandisporum*, *T.pannonicum*), but their phylogenetic and morphological results indicated the presence of at least 19 previously-undescribed European species of *Tulostoma*, nine of which had been collected in Hungary. The species diversity in Eastern Europe was further emphasised by Rusevska et al. (2019) who reported four species from North Macedonia distinct from all known and described species.

**Figure 1.**
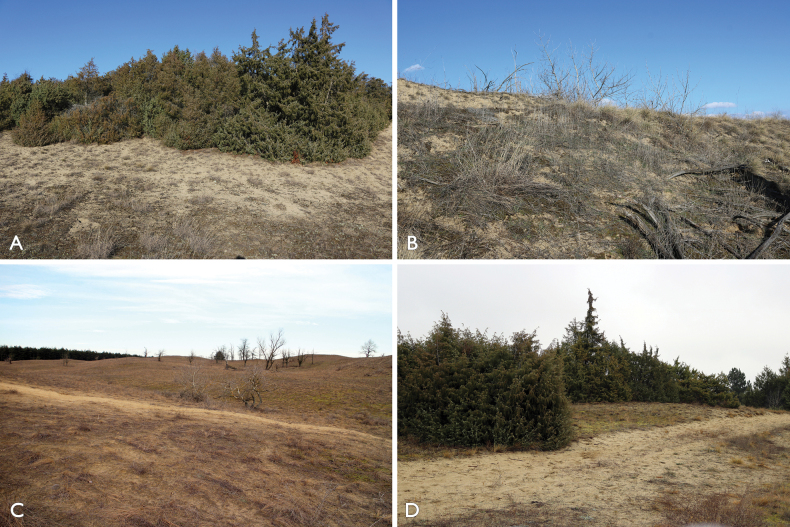
Habitats of *Tulostoma* species in Hungary: **a***T.hungaricum* in Orgovány **b***T.sacchariolens* in Orgovány **c***T.dunense* in Izsák (Soltszentimre) **d***T.shaihuludii* in Izsák (Soltszentimre). Photos: P. Finy.

In Europe, Hungary has an exceptionally large diversity of gasteroid taxa mainly due to the suitable habitats of the Pannonian sandy steppe areas of the country (Fig. 1). The *Festucetumvaginatae* plant communities are characteristic on open, continental sandy soils, dominated by the grass species *Festucavaginata* which also occur on open steppe mosaics between the poplar–juniper sand dune thickets (Bölöni et al. 2011; Rimóczi et al. 2011). Gasteroid fungi occur especially in those areas where *Stipaborysthenica*, *Fumanaprocumbens* or *Juniperuscommunis* are present (Fig. 1).

In this paper, we propose four species of *Tulostoma* new to science, two of which were retrieved previously by Jeppson et al. (2017) as *Tulostoma* sp. 1 and T.aff.cretaceum. Additionally, we present two further species identified through subsequent collections and field investigations. One of the new species was previously reported from Hungary under the name *T.volvulatum* (Hollós 1904; Siller and Vasas 1995; Siller et al. 2005) and *T.obesum* (Siller et al. 2006; Rimóczi et al. 2011) which is listed as a protected species by Hungarian law.

## ﻿Materials and methods

Samples of *Tulostoma* were collected in Hungary over a period of more than 25 years. Collecting has mostly been performed in the sandy habitats of the Great Hungarian Plain on both sides of the Danube (Kiskunság, Mezőföld). Studied collections were deposited in the herbaria BP (only holotypes), GB and in the Department of Plant Anatomy, Eötvös Loránd University (abbreviated further as ELTE).

### ﻿Macromorphological study

Mature basidiomata of *Tulostoma* were collected and studied under a stereomicroscope, regarding their macromorphological characteristics (size, colour, shape of the spore-sac (capitulum), type of mouth (ostiole), type of exoperidium as well as features of the stem), in accordance with Wright (1987). In situ or ex situ photo-documentation of each sample was carried out.

### ﻿Microscopy

Microscopic features were studied under an Olympus BH-2 light microscope. Samples were mounted in lactophenol-cotton blue and heated to boiling temperature. Measurements were performed under 1000× magnification and calculated digitally using Piximètre software (www.piximetre.fr). Spore dimensions are given without the ornamentation of the spore walls. Small pieces of peridium and gleba from dried basidiomata were prepared, fixed to stubs, coated with gold and examined under a Hitachi S2460N (Hitachi Ltd., Tokyo, Japan) scanning electron microscope (SEM) at 22 kV accelerating voltage.

### ﻿Molecular study

Total DNA extraction was carried out with the E.Z.N.A. SP Fungal DNA Mini Kit (Omega Bio-Tek, Norcross, GA, USA) and NucleoSpin Plant II Mini Kit (Macherey-Nagel, Düren, Germany) following the instructions of the manufacturers. The ITS (internal transcribed spacer) region of the nrDNA which is the universal fungal DNA barcode region (Schoch et al. 2012) was amplified using the primer pairs ITS1F/ITS4 (White et al. 1990; Gardes and Bruns 1993) as described in Papp and Dima (2018). The primers LR0R (Rehner and Samuels 1994) and LR5 (Vilgalys and Hester 1990) were used to amplify the partial 28S nrRNA gene (LSU) of the nrDNA operon region. The primers EF1-983F and EF1-2218R (Rehner and Buckley 2005) were used to amplify part of the translation elongation factor 1α (*tef1-α*). Sequencing of the amplicons with the primers used for amplification was carried out by LGC Genomics (Berlin, Germany). The sequences were compiled from electrophoregrams using the Staden software package (Staden et al. 2000) and CodonCode Aligner package (CodonCode Corp., Centerville, Massachusetts, USA). Sequences of each locus (ITS, LSU and *tef1-α*), together with sequences of respective species downloaded from GenBank mainly based on Jeppson et al. (2017), were aligned separately with the online MAFFT version 7 using the E-INS-i strategy (Katoh and Standley 2013) (Table 1). The alignments were checked and edited in MEGA7 (Kumar et al. 2016) and concatenated to one dataset in SeaView 5 (Gouy et al. 2021). Bayesian Inference (BI) analyses were performed with MrBayes 3.1.2 (Ronquist and Huelsenbeck 2003) using a GTR + G substitution model. Four Markov chains were run for 10,000,000 generations, sampling every 1,000 generations with a burn-in value set at 4,000 sampled trees. Maximum Likelihood (ML) phylogenetic analysis was carried out with the raxmlGUI 1.3 implementation (Silvestro and Michalak 2012; Stamatakis 2014). The GTR + G nucleotide substitution model and ML estimation of base frequencies were applied for the partitions. ML bootstrap (BS) analysis with 1,000 replicates was used to test the support of the branches. *Tulostomapulchellum* (MJ7833) and *T.striatum* (Fritz 2010‐2) served as outgroups. Intra- and interspecific genetic differences were calculated by dividing the number of differences (substitutions and/or indels) found in the whole ITS region by the length of the region. Phylogenetic trees were visualised and edited in MEGA 7 (Kumar et al. 2016) and deposited together with the alignments at Figshare repository (10.6084/m9.figshare.24112749). Newly-generated sequences were submitted to GenBank. Studied voucher collections are presented in Table 1.

**Table 1.** Sequences used in this study. Newly-generated sequences are marked in boldface.

Name	Strain/Voucher	Country	ITS	LSU	TEF	References
*Tulostomaahmadii*	HUP SH-33b, holotype	Pakistan	KP738712	–	–	Hussain et al. (2016)
*Tulostomaalbicans*	B2092, P.S. Catcheside 1266	Australia	–	MK278628	–	Varga et al. (2019)
*Tulostomaalbicans*	Cope, NY, Holotype	United States	KX576548	–	–	Jeppson et al. (2017)
*Tulostomabeccarianum*	Finy2	Hungary	KU519076	KU519076	KU843959	Jeppson et al. (2017)
*Tulostomabeccarianum*	Herb. Bresadola (S), holotype	Italy	KX640979	–	–	Jeppson et al. (2017)
*Tulostomaberkeleyi*	JLH MyCoPortal 6604754	United States	MK578704	MK578704	–	Unpublished
*Tulostomabrumale*	Finy9	Hungary	KU519059	KU519059	KU843944	Jeppson et al. (2017)
*Tulostomacalcareum*	Finy4	Hungary	KU519088	KU519088	KU843895	Jeppson et al. (2017)
*Tulostomacalcareum*	MJ6965, holotype	Sweden	KU519086	KU519086	KU843881	Jeppson et al. (2017)
*Tulostomacalongei*	MJ8773, holotype	Spain	KU518973	KU518973	KU844000	Jeppson et al. (2017)
*Tulostomacaespitosum* cf.	SNMH9	Slovakia	MK907419	MK907419	–	Unpublished
*Tulostomacaespitosum* cf.	MJ881114	Spain	KU519031	KU519031	KU843978	Jeppson et al. (2017)
*Tulostomacaespitosum* cf.	AH15040	Spain	KU519032	KU519032	KU843979	Jeppson et al. (2017)
***Tulostomacretaceum***	**NY737977, holotype**	**United States**	**OR722641**	**OR722660**	–	**This study**
*Tulostomacretaceum* cf. 1	Knudsen0107	Russia	KU518993	KU518993	KU843988	Jeppson et al. (2017)
*Tulostomacretaceum* cf. 2	AH13672	Spain	KU518998	KU518998	KU843991	Jeppson et al. (2017)
*Tulostomacretaceum* cf. 2	AH3995	Spain	KU518999	KU518999	KU843992	Jeppson et al. (2017)
*Tulostomacretaceum* cf. 2	MJ6194	Spain	KU518997	KU518997	KU843989	Jeppson et al. (2017)
*Tulostomacretaceum* cf. 2	MJ9304	Spain	KU519000	KU519000	KU843990	Jeppson et al. (2017)
***Tulostomacretaceum* cf. 3**	**FP-2023-05-11-1**	**Kazakhstan**	**OR722639**	**OR722658**	–	**This study**
***Tulostomacretaceum* cf. 3**	**FP-2023-05-11-4**	**Kazakhstan**	**OR722640**	**OR722659**	–	**This study**
*Tulostomacretaceum* cf. 3	SNMH10	Kazakhstan	MK907420	MK907420	–	Unpublished
*Tulostomacretaceum* cf.	MJ3821	Hungary	KU518994	KU518994	KU843993	Jeppson et al. (2017)
*Tulostomacyclophorum*	MJ8862	Hungary	KU518985	KU518985	KU843963	Jeppson et al. (2017)
*Tulostomadomingueziae*	MLHC24 (CORD), holotype	Argentina	HQ667594	HQ667597	–	Caffot et al. (2011)
***Tulostomadunense***	**BP112640, holotype**	**Hungary**	**OR722622**	**OR722648**	**OR707014**	**This study**
***Tulostomadunense***	**DB-2021-11-21-2**	**Hungary**	**OR722626**	–	–	**This study**
***Tulostomadunense***	**FP-2019-12-07**	**Hungary**	**OR722617**	**OR722643**	**OR707009**	**This study**
***Tulostomadunense***	**FP-2020-12-06**	**Hungary**	**OR722618**	**OR722644**	**OR707010**	**This study**
***Tulostomadunense***	**FP-2022-01-02-1**	**Hungary**	**OR722619**	**OR722645**	**OR707011**	**This study**
***Tulostomadunense***	**FP-2021-01-02**	**Hungary**	**OR722620**	**OR722646**	**OR707012**	**This study**
***Tulostomadunense***	**FP-2016-06-05**	**Hungary**	**OR722621**	**OR722647**	**OR707013**	**This study**
***Tulostomadunense***	**FP-2021-02-18**	**Hungary**	**OR722623**	**OR722649**	**OR707015**	**This study**
***Tulostomadunense***	**FP-2015-12-06**	**Hungary**	**OR722624**	**OR722650**	**OR707016**	**This study**
***Tulostomadunense***	**FP-2016-12-11**	**Hungary**	**OR722625**	**OR722651**	**OR707017**	**This study**
*Tulostomadunense*	MJ6103 (as cf.cretaceum)	Hungary	KU518995	KU518995	KU843994	Jeppson et al. (2017)
*Tulostomadunense*	MJ7759 (as cf.cretaceum)	Hungary	KU518996	KU518996	KU843995	Jeppson et al. (2017)
*Tulostomaeckbladii*	Sivertsen930717, TRH9565, holotype	Norway	KU519069	KU519069	KU843952	Jeppson et al. (2017)
*Tulostomaexcentricum*	BPI 729284, holotype	United States	KU519055	KU519055	–	Jeppson et al. (2017)
*Tulostomafimbriatum*	Finy8	Hungary	KU518968	KU518968	KU843912	Jeppson et al. (2017)
*Tulostomafimbriatum*	Månsson 991010, epitype	Sweden	KU518963	KU518963	KU843904	Jeppson et al. (2017)
*Tulostomafulvellum*	Kabát 970428	Slovakia	KU518991	KU518991	KU844001	Jeppson et al. (2017)
*Tulostomagiovanellae*	MJ8706	Spain	KU519071	KU519071	KU843954	Jeppson et al. (2017)
*Tulostomagrandisporum*	Finy10	Hungary	KU519005	KU519005	KU843922	Jeppson et al. (2017)
*Tulostomagrandisporum*	MJ8907, holotype	Hungary	KU519003	KU519003	KU843924	Jeppson et al. (2017)
***Tulostomahungaricum***	**BP112641, holotype**	**Hungary**	**OR722630**	**OR722653**	–	**This study**
***Tulostomahungaricum***	**FP-2019-01-23**	**Hungary**	**OR722627**	–	–	**This study**
***Tulostomahungaricum***	**FP-2021-02-19**	**Hungary**	**OR722628**	–	–	**This study**
***Tulostomahungaricum***	**FP-2022-01-02-2**	**Hungary**	**OR722629**	**OR722652**	**OR707021**	**This study**
*Tulostomakotlabae*	Brůžek 140918	Czech Republic	KU519028	KU519028	KU843977	Jeppson et al. (2017)
*Tulostomakotlabae*	Kotlaba (PRM 704203), holotype	Slovakia	KX576544	KX576544	–	Jeppson et al. (2017)
Tulostomacf.kotlabae	MJ5996	Hungary	KU519016	KU519016	KU843966	Jeppson et al. (2017)
Tulostomacf.kotlabae	Finy1	Hungary	KU519017	KU519017	KU843967	Jeppson et al. (2017)
Tulostomacf.kotlabae	MJ7795	Hungary	KU519020	KU519020	KU843970	Jeppson et al. (2017)
***Tulostomalaceratum***	**NY834492**	**United States**	**OR722642**	**OR722661**	–	**This study**
*Tulostomalloydii*	Lahti 201210	Italy	KU518990	KU518990	KU843965	Jeppson et al. (2017)
*Tulostomalusitanicum*	LISU-MGA-8	Portugal	KX576542	KX576542	–	Jeppson et al. (2017)
*Tulostomalysocephalum*	Long 9639, holotype	United States	KU519034	KU519034	–	Jeppson et al. (2017)
*Tulostomamelanocyclum*	MJ090418	Hungary	KU519106	KU519106	KU843890	Jeppson et al. (2017)
Tulostomacf.nanum	MJ4976	Hungary	KU519036	KU519036	KU843968	Jeppson et al. (2017)
*Tulostomaniveum*	MJ7692	Sweden	KU519078	KU519078	KU843932	Jeppson et al. (2017)
*Tulostomaobesum*	Cooke 2715, isotype	United States	KX576541	KX576541	–	Jeppson et al. (2017)
*Tulostomaobesum*	MJ8695	Spain	KU518986	KU518986	KU843985	Jeppson et al. (2017)
*Tulostomapannonicum*	MJ7764, holotype	Hungary	KU519010	KU519010	–	Jeppson et al. (2017)
*Tulostomapannonicum*	MJ7803	Hungary	KU519011	KU519011	KU843996	Jeppson et al. (2017)
*Tulostomapseudopulchellum*	AH 11603, paratype	Spain	KU519012	KU519012	KU843997	Jeppson et al. (2017)
*Tulostomapseudopulchellum*	AH 11605, holotype	Spain	KX513827	KX513827	–	Jeppson et al. (2017)
*Tulostomapulchellum*	MJ7833	Hungary	KU518957	KU518957	KU843928	Jeppson et al. (2017)
*Tulostomapunctatum*	BPI 729033, lectotype	United States	KC333071	KC333071	–	Jeppson et al. (2017)
*Tulostomapunctatum*	MJ7472	Slovakia	KU518952	KU518952	KU843875	Jeppson et al. (2017)
*Tulostomapygmaeum* cf.	Brůžek 131207	Slovakia	KU519041	KU519041	KU843931	Jeppson et al. (2017)
*Tulostomarufum*	BPI 704578, holotype	United States	KU519107	KU519107	–	Jeppson et al. (2017)
***Tulostomasacchariolens***	**BP112642, holotype**	**Hungary**	**OR722632**	**OR722654**	**OR707020**	**This study**
***Tulostomasacchariolens***	**FP-2019-12-06**	**Hungary**	**OR722631**	–	–	**This study**
***Tulostomasacchariolens***	**FP-2021-01-24b**	**Hungary**	**OR722633**	–	–	**This study**
***Tulostomasacchariolens***	**FP-2021-02-18**	**Hungary**	**OR722634**	**OR722655**	–	**This study**
***Tulostomashaihuludii***	**BP112643, holotype**	**Hungary**	**OR722637**	**OR722657**	**OR707019**	**This study**
***Tulostomashaihuludii***	**FP-2020-12-01**	**Hungary**	**OR722635**	–	–	**This study**
***Tulostomashaihuludii***	**FP-2020-12-27**	**Hungary**	**OR722636**	**OR722656**	**OR707018**	**This study**
***Tulostomashaihuludii***	**FP-2017-12-09**	**Hungary**	**OR722638**	–	–	**This study**
*Tulostomashaihuludii*	MJ7762	Hungary	KU518979	KU518979	KU843981	Jeppson et al. (2017)
*Tulostomasimulans*	MJ3844	Hungary	KU519052	KU519052	KU843941	Jeppson et al. (2017)
*Tulostoma* sp. 10	MJ3813	Hungary	KU519029	KU519029	–	Jeppson et al. (2017)
*Tulostoma* sp. 14	MJ5004	Spain	KU519039	KU519039	KU843999	Jeppson et al. (2017)
*Tulostoma* sp. 20	MJ5015	Spain	KU519067	KU519067	KU843950	Jeppson et al. (2017)
*Tulostoma* sp. 21	AH11698	Spain	KX640986	KX640986	–	Jeppson et al. (2017)
*Tulostomasquamosum*	Larsson 260-06	France	KU519097	KU519097	KU843892	Jeppson et al. (2017)
*Tulostomastriatum*	Fritz 2010-2	Mongolia	KU518958	KU518958	KU843929	Jeppson et al. (2017)
*Tulostomasubmembranaceum*	AH15132, holotype	Mexico	KX513826	KX513826	–	Jeppson et al. (2017)
*Tulostomasubmembranaceum* cf.	MJ9296	Spain	KU519014	KU519014	KU843984	Jeppson et al. (2017)
*Tulostomasubsquamosum*	MJ4945	Hungary	KU519091	KU519091	KU843899	Jeppson et al. (2017)
*Tulostomaverrucosum*	CCB142	United States	MG663293	MG663293	–	Unpublished
*Tulostomawinterhoffii*	MJ7761	Hungary	KU518976	KU518976	KU843916	Jeppson et al. (2017)
*Tulostomaxerophilum*	Long 9688, BPI 802484, holotype	United States	KX576549	–	–	Jeppson et al. (2017)

## ﻿Results

### ﻿Phylogenetic analysis

**Figure 2.**
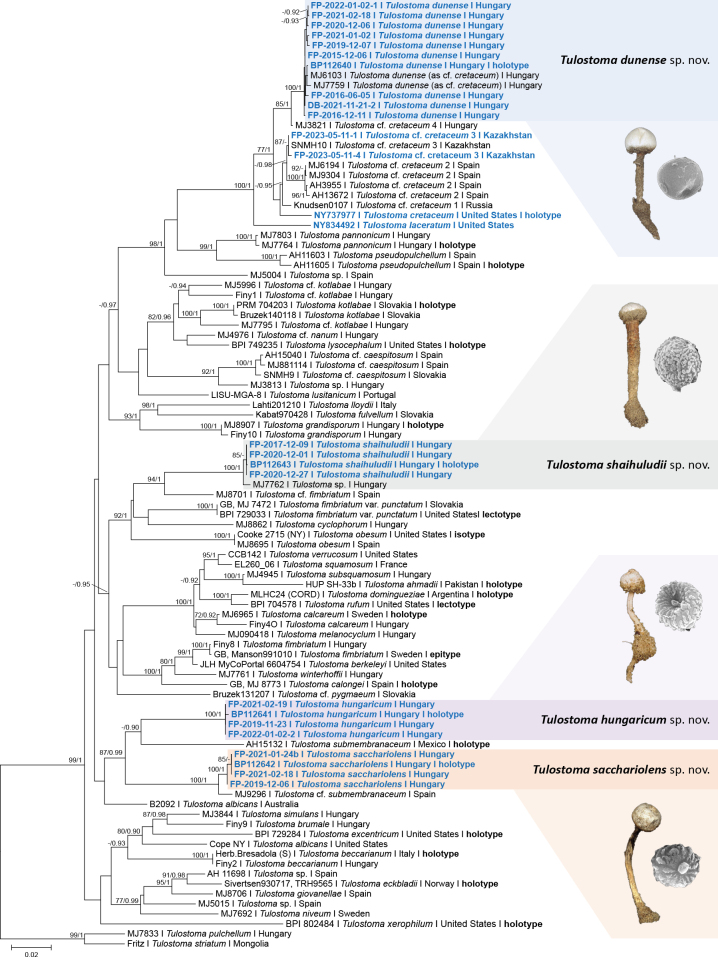
Maximum Likelihood (RAxML) tree of concatenated nrDNA ITS, nrDNA LSU and *tef1-α* sequences of representative species of the genus *Tulostoma* and the four newly-introduced species in the present study. Sequences obtained in this study are shown in bold blue. After the voucher number, the species and the country of origin are shown. Then, the type specimens are indicated. ML bootstrap support values (≥ 70) are shown before slashes and Bayesian posterior probabilities (≥ 0.90) are shown after slashes. Highlighted sections indicate affiliations to the four novel *Tulostoma* species: *T.dunense*, *T.hungaricum*, *T.sacchariolens* and *T.shaihuludii*. The illustrations exhibit basidiomata and basidiospore characteristics of the novel species. *Tulostomapulchellum* (MJ7833) and *T.striatum* (Fritz) served as multiple outgroups. The scale bar indicates expected changes per site per branch.

The three-locus molecular phylogenetic analyses of the newly-generated and representative *Tulostoma* sequences were based on 94 ITS, 76 LSU and 60 *tef1-α* (Table 1) and 3321 characters. In this study, 26 ITS, 26 LSU and 13 *tef1-α* sequences were newly gained, including the ITS and LSU sequences of the holotype of *Tulostomacretaceum* (Table 1). Phylogenetic trees from ML and BI analyses showed congruent topologies and the sequences representing the four new species proposed here formed strongly-supported clades (MLBS/BIPP = 100%/1.00). The best scoring ML tree is shown in Fig. 2.

### ﻿Taxonomy

#### ﻿ Tulostoma dunense

Taxon classificationFungiAgaricalesAgaricaceae

Finy, Jeppson, L. Albert, Ölvedi, Dima & V. Papp sp. nov.

CD5353AA-1FD0-5BAE-B0BF-E60F6329FBD3

MB 849931

Fig. 3

##### Holotype.

Hungary, Tolna, Németkér, open sandy grassland, 18 Oct 2020, P. Finy, I. Ölvedi, FP-2020-10-18 (BP112640, isotype GB). GenBank: ITS OR722622, LSUOR722648, tef1 OR707014.

##### Etymology.

The epithet refers to the continental, open, bare sandy habitat of this species, similar to coastal dunes.

##### Description.

Spore-sac subglobose, depressed-globose, 10–20 mm. Exoperidium hyphal, encrusting sand only at the base of the spore-sac. Endoperidium tough, chalky white or dirty-dingy white, with age becoming greyish, young basidiomata with velvety surface. Mouth prominent, fibrillose-lacerate, irregular sometimes remains unopened for a long time and splits later due to mechanical pressure (wind or trampling). Socket distantly separated from the stem. Stem 35–80 × 1.5–5 mm, initially white, then ochraceous, with age greyish–blackish, longitudinally furrowed, at the base with a volva and a prominent, easily broken pseudorhiza. Gleba ferruginous to brick-red brown, usually scattered on the surface of the spore-sac. Capillitium brown, 2.5–10 µm in diameter with walls 0.7–2.5 µm in diameter, fragile, breaking up at septal levels in 40–350 µm long segments with rounded, not widened ends, rarely branching. Spores subglobose to oval, 4.6–5.2 × 4.0–4.8 µm (av. 4.4 × 4.9 µm), smooth under LM and SEM.

##### Habitat and distribution.

The psammophilous species *Tulostomadunense* known so far only from sandy areas of the Great Hungarian Plain of Hungary. It occurs on both sides of the Danube (Kiskunság, Dél-Mezőföld), where open dunes appear. It mainly grows solitary, deep in the sand in large, open sandy areas to bare spots.

**Figure 3.**
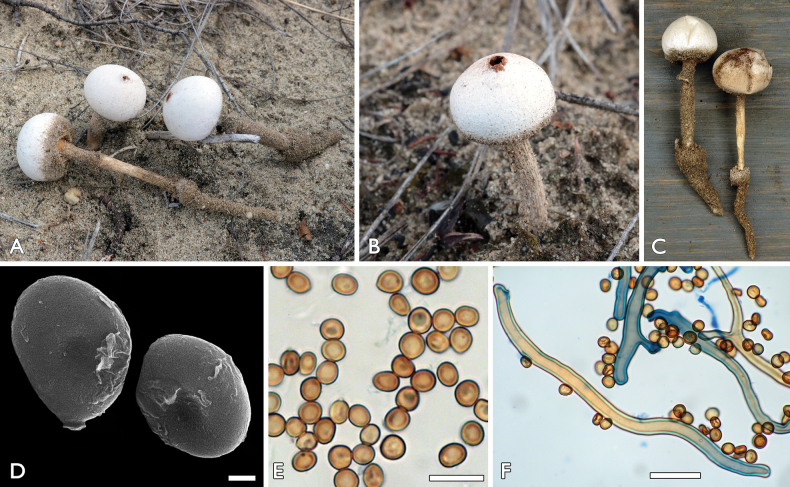
*Tulostomadunense*: **a, c–f** FP-2020-10-18 (BP112640, holotype), Németkér **b** FP-2021-01-02, Tázlár **a–c** basidiocarps **d, e** basidiospores **f** capillitium with basidiospores. Scale bars: 1 μm (**d**); 10 μm (**e**); 10 µm (**f**). Photos: **a, b, e, f** P. Finy **c** L. Albert **d** K. Bóka.

##### Notes.

*Tulostomadunense* was previously recorded in Hungary by Hollós (1904), Siller and Vasas (1995), Babos (1999), Siller et al. (2005), Siller et al. (2006) and Rimóczi et al. (2011) under the names of *T.volvulatum*, *T.obesum* and T.aff.cretaceum. Hollós (1904) included both *T.giovanellae* and *T.dunense* under the name *T.volvulatum* (nom. rej., Altés et al. (1999)) and recorded it in urban places in the City of Kecskemét (now *T.giovanellae*) as well as in sand dunes (now the new species, *T.dunense*). The brownish colour of the capillitium characteristic of specimens from sand dunes and largely absent in those from urban habitats, was considered a result of the maturation process. Later (Hollós 1913) corrected his earlier concept and concluded that some of the synonyms he had listed under *T.volvulatum* in his publication (Hollós 1904), in fact belonged to a complex of several species. He added illustrations of their different types of capillitia (Hollós 1913: tables 3 and 4) and concluded that his concept of *T.volvulatum* from the sand dunes was a synonym of *T.kansense*, a smooth-spored species with brownish capillitium described from North America. The capillitium in the samples from urban habitats clearly showed the undulating inner walls of the capillitium typical of *T.giovanellae* (Hollós 1913: table 3, fig. 6), although Hollós did not identify them under this name. Some 50 years later, Nagy and Babos (1969) recorded *T.giovanellae* growing on a pavement at the base of a house wall in Budapest. It matched partly the material cited by Hollós (1904) as *T.volvulatum*, but was decidedly different from the species growing in the sand dunes. Babos (1999) later came to the same conclusion. Altés et al. (1999) studied the holotype material of *T.volvulatum* and concluded that it was a synonym of *T.giovanellae* characterised by ornamented spores. They also studied the holotype material of *T.obesum* with which they identified European collections with completely smooth spores from steppe habitats and the name *T.volvulatum* was rejected. Rimóczi et al. (2011) accordingly identified the Hungarian species of the sand dunes as *T.obesum*. Molecular data (Jeppson et al. 2017) later showed that the Hungarian “*T.obesum*” was not identical with the American holotype of *T.obesum*, but was closely related to another American species described as *T.cretaceum*. It was recovered as T.aff.cretaceum by Jeppson et al. (2017). The T.aff.cretaceum from Hungary belongs to a complex of cryptic species with a strong geographical isolation. The type of *T.cretaceum* was studied and successfully sequenced by Gube (2009), but the ITS and LSU sequences have remained unpublished. We have kindly received these sequences from Matthias Gube allowing us to include them in the phylogenetic analyses. The phylogenetic analyses showed that the type of *T.cretaceum* formed a distinct lineage within this complex (Fig. 1), proving that this North American species is different from the European and Asian lookalikes. Therefore, the Hungarian collections are proposed here as a novel species, *T.dunense*, which is closely related to samples of T.aff.cretaceum collected in Hungary, Kazakhstan and in the Russian Federation as well as in Spain (Fig. 1). The main features to distinguish *T.dunense* from the other species in the complex are mainly phylogenetic- and geographical-based data. *Tulostomadunense* has been a protected species under Hungarian law since 2005, but to date, it has erroneously been treated under various misinterpreted and dubious names, i.e. *T.volvulatum*, *T.obesum* and T.aff.cretaceum. The ITS region of *T.dunense* differs from its closest clade represented by a single sequence (T.cf.cretaceum MJ3821, see Fig. 2) by at least 13 substitution and indel positions, which is a similarity of 98%. This sequence might represent a different species, but further collections need to be studied to clarify its taxonomic status. In contrast, low intraspecific genetic variation was detected in *T.dunense* (0–4 substitution and indel positions). The ITS and LSU sequences of an old collection identified by Long (www.mycoportal.org) under the name *Schizostomalaceratum* (NY834492) collected in 1941 in New Mexico, were provided for us by Matthias Gube. Our phylogenetic analyses indicate that this specimen belongs to the *T.cretaceum* complex as a distinct lineage. On the other hand, the nomenclature of the genus *Schizostoma*, as well as the species *S.laceratum* (Fries 1829; Léveillé 1846), seems to be problematic and needs further clarification.

##### Specimens examined.

Hungary, Bács-Kiskun, Ágasegyháza, in open sand, 18 Feb 2021, P. Finy, FP-2021-02-18 (ELTE); Bócsa, in open sand, 7 Dec 2019, P. Finy, FP-2019-12-07 (ELTE); Fülöpháza, 11 Apr 2006, T. Knutsson, T. Gunnarsson, J. Jeppson, M. Jeppson, MJ7759 (GB), Ibidem, in open sand, 5 Jun 2016, P. Finy, FP-2016-06-05 (ELTE); Izsák (Soltszentimre), in open sand, 21 Nov 2019, A. Nagy, B. Dima, DB-2021-11-21-2 (ELTE); Kéleshalom, in open sand, 6 Dec 2015, P. Finy, FP-2015-12-06 (ELTE); Ibidem, in open sand, 2 Jan 2022, P. Finy, I. Ölvedi, FP-2022-01-02-1 (ELTE); Tázlár, in open sand, 11 Dec 2016, P. Finy, FP-2016-12-11 (ELTE); Ibidem, in open sand, 2 Jan 2021, P. Finy, FP-2021-01-02 (ELTE). Pest, Örkény, former military training field, sand steppe vegetation, in open sand, 5 Nov 2001, J. Jeppson, M. Jeppson, MJ6103 (GB), Ibidem, in open sand, 6 Dec 2020, P. Finy, L. Albert, FP-2020-12-06 (ELTE).

#### ﻿ Tulostoma hungaricum

Taxon classificationFungiAgaricalesAgaricaceae

Finy, Jeppson, L. Albert, Ölvedi & Dima sp. nov.

56D1B89E-9B0A-57C4-8D79-80023FE5654B

MB 849932

Fig. 4

##### Holotype.

Hungary, Bács-Kiskun, Bócsa, open sandy grassland, on sandy sites with scattered vegetation, near *Juniperuscommunis* shrubs 24 Jan 2021, P. Finy, FP-2021-01-24a (BP112641, isotype GB). GenBank: ITS OR722630, LSUOR722653.

##### Etymology.

With reference to Hungary where it was discovered.

##### Description.

Spore-sac subglobose, 3–6 mm. Exoperidium hyphal, heavily encrusting sand grains. Endoperidium white, pitted from adhering sand grains. Mouth small, fibrillose- fimbriate with a small and inconspicuous mouth. Socket inconspicuous. Stem slender, 9–15 × 1–1.5 mm, whitish, not bulbous. Gleba ochraceous brown. Capillitium elastic, 2–6 µm in diameter with walls 0.5–2 µm in diameter and moderate branching. Septa in general not widened. Basidiospores subglobose, 4.9–5.7 × 4.5–5.1 µm (av. 5.2 × 4.8 µm), varied in size, with fine, but visible ornamentation. SEM-photos show low verrucae coalescing in short lines.

**Figure 4.**
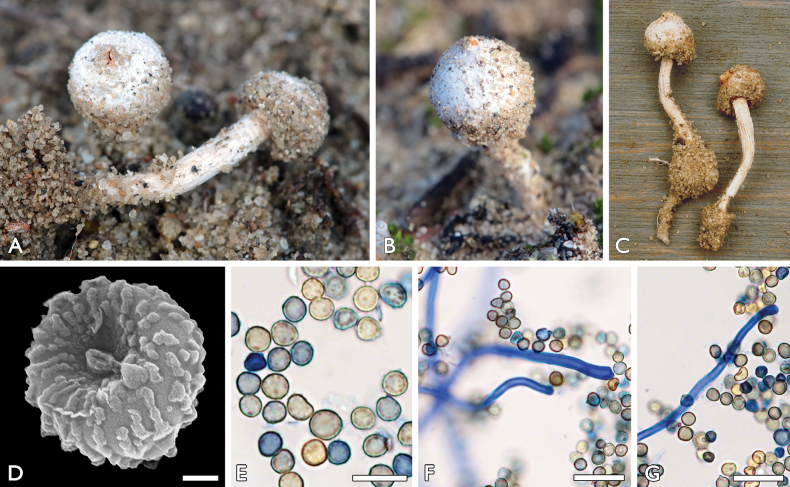
*Tulostomahungaricum*: **a, c–g** FP-2021-01-24a (BP112641, holotype), Bócsa **b** FP-2019-11-23, Orgovány **a–c** basidiocarps **d, e** basidiospores **f, g** capillitium with basidiospores. Scale bars: 1 µm (**d**); 10 µm (**e**); 20 µm (**f, g**). Photos: **a, b, e–g** P. Finy **c** L. Albert **d** K. Bóka.

##### Habitat and distribution.

*Tulostomahungaricum* occurs in the calcareous, sandy steppe areas, in dry and exposed habitats on bare sand. It has, to date, been found on the sheltered and sun-exposed, extremely warm sandy spots on the south-facing sides of *Juniperuscommunis*. So far, it has only been found in few localities of the Kiskunság National Park, Central Hungary.

##### Notes.

*Tulostomahungaricum* is the smallest *Tulostoma* species in Europe. It sometimes shares its habitat with *T.pannonicum*, another species forming small basidiomata. The latter is, however, easily distinguished on its ochraceous stem, membranous exoperidium and smaller spores. *Tulostomahungaricum* is an isolated species belonging to the well-supported Clade 7 according to Jeppson et al. (2017), together with *T.submembranaceum* from Mexico, T.cf.submembranaceum from Spain and the below-described new species *T.sacchariolens*. In the ITS region, *T.hungaricum* differs from its closest species (*T.submembranaceum*, see Fig. 1) by almost 90 substitution and indel positions, which is a similarity of 87%. Low intraspecific genetic variability was observed in *T.hungaricum* by a difference of 0–3 substitution and indel positions.

##### Specimens examined.

Hungary, Bács-Kiskun, Kéleshalom, open sandy grassland, near *Juniperuscommunis*, 2 Jan 2022, P. Finy, I. Ölvedi, L. Albert, FP-2022-01-02-2 (ELTE); Orgovány, open sandy grassland, near *Juniperuscommunis*, 23 Nov 2019, P. Finy, I. Ölvedi, L. Albert, FP-2019-11-23 (ELTE); Ibidem, open sandy grassland, near *Juniperuscommunis*, 19 Feb 2021, P. Finy, I. Ölvedi, L. Albert, FP-2021-02-19 (ELTE).

##### Morphologically examined specimens.

Hungary, Bács-Kiskun, Bócsa, open sandy grassland, near *Juniperuscommunis*, 3 Dec 2022, P. Finy, I. Ölvedi, L. Albert, FP-2022-12-03 (herb. Finy); Fülöpháza, open sandy grassland, near *Juniperuscommunis*, 14 Jan 2023, P. Finy, I. Ölvedi, L. Albert, FP-2023-01-14 (herb. Finy); Pest, Tatárszentgyörgy, open sandy grassland, near *Juniperuscommunis*, 17 Dec 2022, I. Ölvedi, OP-2022-12-17 (herb. Ölvedi).

#### ﻿ Tulostoma sacchariolens

Taxon classificationFungiAgaricalesAgaricaceae

Finy, Jeppson, L. Albert, Ölvedi & Dima sp. nov.

ADB45650-884B-59A1-88D4-32B55EFC27B8

MB 849933

Fig. 5

##### Holotype.

Hungary, Bács-Kiskun, Bócsa, open disturbed sandy grassland, in a sand pit, on bare ground, 24 Jan 2021, I. Ölvedi, P. Finy, L. Albert, OP20210124 (BP112642, isotype GB). GenBank: ITS OR722632, LSUOR722654, tef1 OR707020.

##### Etymology.

The epithet refers to its unique sweetish floral smell reminiscent of that of, for example, *Hebelomasacchariolens*.

##### Description.

Spore-sac subglobose, often flattened to depressed or hemispherical, 5–9 mm. Exoperidium hyphal, heavily encrusting sand, more persistent at the base of the spore-sac. Endoperidium white or dirty white, pitted from detached sand grains. Mouth delicately fimbriate. Socket conspicuous, forming a thickening on the upper part of the stem. Stem 25–50 × 1.5–2.5 mm, whitish, ornamented with orange to reddish fibrils, with age remarkably blackening, thickening towards the base, bulbous. The mature basidiomata have a pronounced sweet floral smell, reminiscent of *Hebelomasacchariolens* Quél. or *Freesia* flowers. Gleba ferruginous brown. Capillitium 2.5–7 µm in diameter with walls 0.8–2.2 µm in diameter, lumen in general scarce, mostly straight, little branching. Most septa slightly widened. Spores subglobose, 4.6–5.3 × 4.1–5 µm (av. 4.6–4.9 µm), with coarse elongated ornamentation. SEM-photos show developed crests arranged in lines.

**Figure 5.**
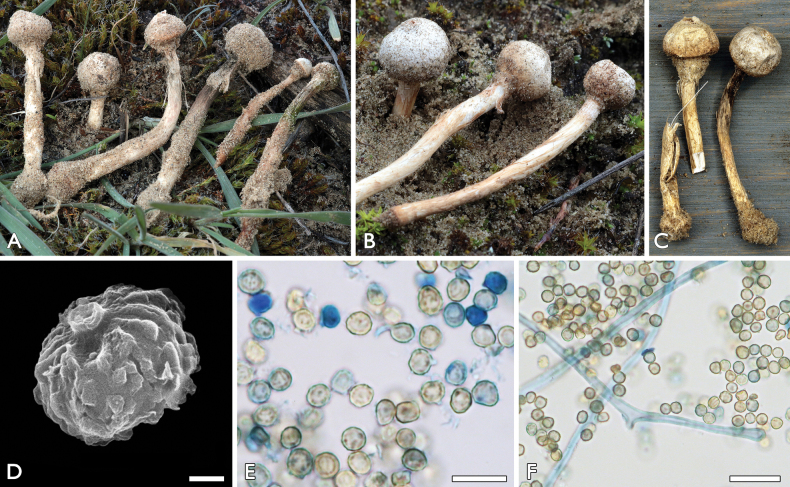
*Tulostomasacchariolens*: **a, e** FP-2021-02-18, Orgovány **b** FP-2019-12-06, Bócsa **c, d, f** OP-2021-01-24 (BP112642, holotype), Bócsa **a–c** basidiocarps **d, e** basidiospores **f** capillitium with basidiospores. Scale bars: 1 µm (**d**); 10 µm (**e**); 20 µm (**f**). Photos: **a, b, e, f** P. Finy **c** L. Albert **d** K. Bóka.

##### Habitat and distribution.

Recorded in calcareous, sandy steppe areas, mostly in sunny open habitats with sparse vegetation, often in trampled or otherwise disturbed places. It is currently known only from a few localities in the sandy areas of the Danube–Tisza interfluves in Central Hungary.

##### Notes.

With its fragrant smell and blackening stem, *Tulostomasacchariolens* has a unique combination of characters within the genus, easily separating it from any known *Tulostoma* species. *Tulostomasacchariolens* belongs to Clade 7 according to Jeppson et al. (2017) together with T.cf.submembranaceum (MJ9296, see Fig. 2) from Spain, *T.submembranaceum* from Mexico and the above-described *T.hungaricum*. It differs from its sister species (T.cf.submembranaceum) in the ITS region by more than 20 substitution and indel positions, which is a similarity of 96%. The intraspecific genetic variability in the ITS region amongst three sequences of *T.sacchariolens* was zero (Fig. 1), while the ITS sequence of FP-2019-12-06 had six polymorphic sites.

##### Specimens examined.

Hungary, Bács-Kiskun: Bócsa, open sandy grassland, 6 Dec 2019, P. Finy, FP-2019-12-06 (ELTE); Ibidem, open sandy grassland, 24 Jan 2021, P. Finy, I. Ölvedi, L. Albert, FP-2021-01-24b (ELTE); Orgovány, open sandy grassland, 18 Feb 2021, P. Finy, I. Ölvedi FP-2021-02-18 (ELTE).

##### Morphologically examined specimens.

Hungary, Bács-Kiskun: Bócsa, open sandy grassland, 3 Dec 2022, P. Finy, FP-2022-12-03 (herb. Finy); Fülöpháza, open sandy grassland, 14 Jan 2023, P. Finy, FP-2023-01-14 (herb. Finy); Orgovány, open sandy grassland, 4 Dec 2021, P. Finy, I. Ölvedi, L. Albert, FP-2021-12-04 (herb. Finy); Pest, Örkény, open sandy grassland, 12 Jan 2022, I. Ölvedi, OP-2022-01-12 (herb. Ölvedi); Ibidem, open sandy grassland, 10 Dec 2022, P. Finy, I. Ölvedi, L. Albert, FP-2022-12-10 (herb. Finy).

#### ﻿ Tulostoma shaihuludii

Taxon classificationFungiAgaricalesAgaricaceae

Finy, Jeppson, L. Albert, Ölvedi, D.G. Knapp & Dima sp. nov.

A4ABA9DA-2478-5E5B-BBA0-44DAF6B260E7

MB 849934

Fig. 6

##### Holotype.

Hungary, Bács-Kiskun, Tázlár, open sandy grassland, 11 Dec 2016, P. Finy, FP-2016-12-11 (BP112643, isotype GB). GenBank: ITS OR722637, LSUOR722657, tef1 OR707019.

##### Etymology.

The epithet refers to its being reminiscent of the sandworm Shai-Hulud of the fictional planet Arrakis from the science fiction novel series Dune by Frank Herbert.

##### Description.

Spore-sac subglobose, often flattened to depressed, 7–18 mm, relatively small compared to the size of the stem. Exoperidium hyphal, encrusting sand at the base of the spore-sac. Endoperidium white or greyish-white, pitted from detached sand grains. Mouth fimbriate, somewhat prominent. Socket developed, slightly separated from the stem. The spore-sac rarely detaches from the stem. Stem 30–70 × 3–6 mm, yellowish-brown to orange brown or reddish-brown, with age darkening, longitudinally furrowed, scaly, often curved, at the base slightly bulbous, with a conspicuous, but fragile pseudorhiza. Gleba ferruginous-cinnamon-brown. Capillitium 3.5–7 µm in diameter with walls 0.3–3.2 µm in diameter, mostly straight, sparsely branched, inner wall often undulating. Septa not or slightly widened. Abundant, thin-walled, septate capillitium hyphae present amongst normal capillitium threads. Basidiospores globose, sometimes flattened, 4.1–5.2 × 3.5–4.7 µm (av. 4.1 × 4.6 µm), finely asperulate, ornamentation not always visible under LM. SEM photos show fine warts arranged in lines forming a dense network.

**Figure 6.**
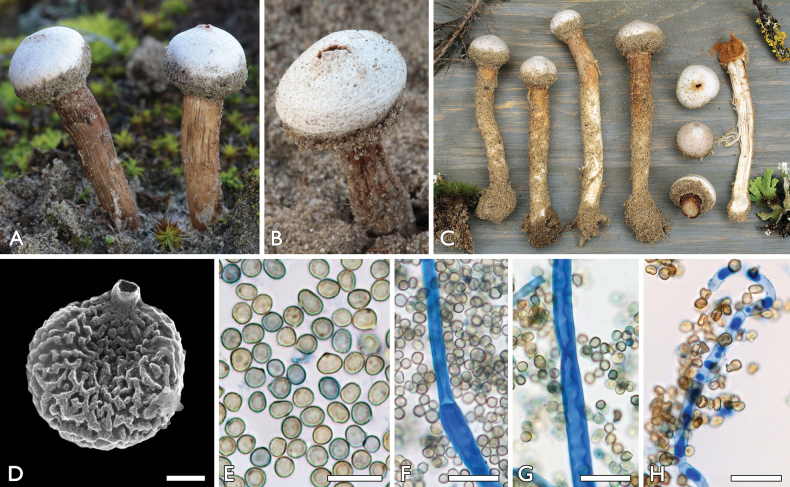
*Tulostomashaihuludii*: **a** FP-2020-12-01-3, Fülöpháza **b, d–g** FP-2016-12-11 (BP112643, holotype), Tázlár **c** AL-2021-01-24, Bócsa **h** FP-2017-12-09, Orgovány **a–c** basidiocarps **d, e** basidiospores **f, g** capillitium with basidiospores **h** thin-walled, septate capillitium hyphae. Scale bars: 1 µm (**d**); 10 µm (**e**); 20 µm (**f–h**). Photos: **a, b, e–h** P. Finy **c** L. Albert **d** K. Bóka.

##### Habitat and distribution.

Occurs in dry and loose calcareous, open sandy habitats of the *Festucetumvaginatae* natural grasslands. It mainly grows solitary, deeply rooted in the sand in spots with bare sand. It is currently known only from the sandy areas of Central Hungary.

##### Notes.

*Tulostomashaihuludii* is similar in stature to *T.fimbriatum* and *T.winterhoffii*, but can be easily distinguished by its habitat (open sand) and its microcharacters, particularly the spore wall ornamentation. It belongs to Clade 2 according to Jeppson et al. (2017) and it forms a sister clade of Tulostomacf.fimbriatum (MJ8701 as “*T.* sp2” in Jeppson et al. (2017)) from which it differs in the ITS region by 45 substitution and indel positions, which is a similarity of 93%. The intraspecific genetic variability of *T.shaihuludii* is low (0–3 substitution and indels positions).

##### Specimens examined.

Hungary, Bács-Kiskun, Fülöpháza, Fülöpházi homokbuckák, sand steppe vegetation, 11 Apr 2006, J. Jeppson, M. Jeppson, MJ7762 (GB); Ibidem, open sandy grassland, 1 Dec 2020, P. Finy, I. Ölvedi, FP-2020-12-01-3 (ELTE); Orgovány, open sandy grassland, 9 Dec 2017, P. Finy, FP-2017-12-09 (ELTE); Pirtó, open sandy grassland, 27 Dec 2020, P. Finy, L. Albert, FP-2020-12-27 (ELTE).

##### Morphologically examined specimens.

Hungary, Bács-Kiskun, Bócsa, open sandy grassland, 7 Dec 2019, P. Finy, FP-2019-12-07 (herb. Finy); Ibidem, open sandy grassland, 24 Jan 2021, P. Finy, L. Albert, I. Ölvedi, FP-2021-01-24 (herb. Finy), AL-2021-01-24 (herb. Albert); Ibidem, open sandy grassland, 4 Dec 2021, P. Finy, I. Ölvedi, FP-2021-12-04 (herb. Finy); Ibidem, open sandy grassland, 3 Dec 2022, P. Finy, FP-2022-12-03 (herb. Finy); Fülöpháza, open sandy grassland, 2 Dec 2018, P. Finy, FP-2018-12-02 (herb. Finy); Ibidem, open sandy grassland, 16 Jan 2022, P. Finy, I. Ölvedi, FP-2022-01-16 (herb. Finy); Izsák (Soltszentimre), open sandy grassland, 4 Feb 2016, P. Finy, FP-2016-02-04 (herb. Finy); Ibidem, open sandy grassland, 14 Dec 2016, P. Finy, FP-2016-12-14 (herb. Finy); Ibidem, open sandy grassland, 17 Jan 2019, P. Finy, FP-2019-01-17 (herb. Finy); Ibidem, open sandy grassland, 16 Dec 2020, P. Finy, FP-2020-12-16-1 (herb. Finy); Kéleshalom, open sandy grassland, 6 Dec 2015, P. Finy, FP20151206 (herb. Finy); Ibidem, open sandy grassland, 2 Jan 2022, P. Finy, I. Ölvedi, FP-2022-01-02-3 (herb. Finy); Kiskunhalas, open sandy grassland, 22 Dec 2019, P. Finy, FP-2019-12-22 (herb. Finy); Ibidem, open sandy grassland, 5 Jan 2023, P. Finy, I. Ölvedi, FP-2023-01-05 (herb. Finy); Kunbaracs, open sandy grassland, 5 Feb 2022, P. Finy, I. Ölvedi, FP-2022-02-05 (herb. Finy); Orgovány, open sandy grassland, 13 Aug 2017, P. Finy, FP-2017-08-13 (herb. Finy); Ibidem, open sandy grassland, 18 Feb 2021, P. Finy, I. Ölvedi, FP-2021-02-18 (herb. Finy); Pirtó, open sandy grassland, 16 Jan 2016, P. Finy, FP-2016-01-16 (herb. Finy); Tázlár, open sandy grassland, 11 Dec 2016, P. Finy, FP-2016-12-11 (herb. Finy); Pest, Örkény, open sandy grassland, 12 Jan 2022, I. Ölvedi, OP-2022-01-12 (herb. Ölvedi); Tatárszentgyörgy, open sandy grassland, 1 Jan 2022, I. Ölvedi, OP-2022-01-01 (herb. Ölvedi); Ibidem, open sandy grassland, 10 Dec 2022, P. Finy, FP-2022-12-10 (herb. Finy); Ibidem, open sandy grassland, 17 Dec 2022, I. Ölvedi, OP-2022-12-17 (herb. Ölvedi); Tolna, Paks, open sandy grassland, 4 Feb 2018, P. Finy, FP-2018-02-04 (herb. Finy); Ibidem, open sandy grassland, 22 Jan 2021, P. Finy, FP-2021-01-22 (herb. Finy); Ibidem, open sandy grassland, 9 Jan 2022, I. Ölvedi, P. Finy, OP-2022-01-09 (herb. Ölvedi); Ibidem, open sandy grassland, 27 Feb 2022, P. Finy, FP-2022-02-27 (herb. Finy).

## ﻿Discussion

The results of our study further emphasise the high species diversity amongst the stalked puffballs (*Tulostoma*) in East Central Europe, as previously indicated by Jeppson et al. (2017). In Hungary, so far 19 species have been recorded, including the four new species proposed in this study. The Pannonian, dry and sandy grasslands between the rivers Danube and Tisza, as well as adjacent areas in Central Hungary, harbour to date 66% of all described species of *Tulostoma* known to occur in Europe (29 spp.). The dry, sandy grasslands in Central Hungary have a long continuity as natural grasslands or as sheep pastures and are characterised by steppe flora and fauna. Both natural and grazed habitats are rich in gasteroid fungi, but usually their species composition is different. The summer and autumn temperatures in the sand are extremely high and the yearly precipitation is low. The dry and drought-resisting basidiomata of *Tulostoma* species could be considered as adaptations to xeric conditions. The development of the basidiomata occurs mainly in late autumn and early winter.

*Tulostoma* species are generally rare (although locally abundant) and the current knowledge of their population structures in Europe is limited. However, their occurrences are highly dependent on the habitat status where they grow and changes in land management are likely to be detrimental to their populations. A vast majority of the European *Tulostoma* species are Red-Listed in the countries where they occur (http://www.eccf.eu/redlists-en.ehtml).

In addition to the four novel species proposed herein, the results from previous works (e.g. Jeppson et al. (2017)) and our ongoing studies indicate the presence of many more undescribed species of *Tulostoma* in Central Europe.

## Supplementary Material

XML Treatment for Tulostoma dunense

XML Treatment for Tulostoma hungaricum

XML Treatment for Tulostoma sacchariolens

XML Treatment for Tulostoma shaihuludii

## ﻿Additional information

### Author ORCIDs

Dániel G. Knapp https://orcid.org/0000-0002-7568-238X

Viktor Papp https://orcid.org/0000-0001-6994-8156

Károly Bóka https://orcid.org/0000-0002-1324-3592

Gábor M. Kovács https://orcid.org/0000-0001-9509-4270

Bálint Dima https://orcid.org/0000-0003-2099-3903

### Data availability

All the data that support the findings of this study are available in the main text or in publicly accessible data repositories, as indicated in the text.
